# Eco-Metabolomics and Metabolic Modeling: Making the Leap From Model Systems in the Lab to Native Populations in the Field

**DOI:** 10.3389/fpls.2018.01556

**Published:** 2018-11-06

**Authors:** Matthias Nagler, Thomas Nägele, Christian Gilli, Lena Fragner, Arthur Korte, Alexander Platzer, Ashley Farlow, Magnus Nordborg, Wolfram Weckwerth

**Affiliations:** ^1^Department of Ecogenomics and Systems Biology, University of Vienna, Vienna, Austria; ^2^LMU Munich, Plant Evolutionary Cell Biology, Munich, Germany; ^3^Vienna Metabolomics Center (VIME), University of Vienna, Vienna, Austria; ^4^Center for Computational and Theoretical Biology, University of Würzburg, Würzburg, Germany; ^5^Gregor Mendel Institute of Molecular Plant Biology, Austrian Academy of Sciences, Vienna, Austria

**Keywords:** eco-metabolomics, *in situ* analysis, metabolomics, metabolic modeling, SNP, natural variation, Jacobian matrix, green systems biology

## Abstract

Experimental high-throughput analysis of molecular networks is a central approach to characterize the adaptation of plant metabolism to the environment. However, recent studies have demonstrated that it is hardly possible to predict *in situ* metabolic phenotypes from experiments under controlled conditions, such as growth chambers or greenhouses. This is particularly due to the high molecular variance of *in situ* samples induced by environmental fluctuations. An approach of functional metabolome interpretation of field samples would be desirable in order to be able to identify and trace back the impact of environmental changes on plant metabolism. To test the applicability of metabolomics studies for a characterization of plant populations in the field, we have identified and analyzed *in situ* samples of nearby grown natural populations of *Arabidopsis thaliana* in Austria. *A. thaliana* is the primary molecular biological model system in plant biology with one of the best functionally annotated genomes representing a reference system for all other plant genome projects. The genomes of these novel natural populations were sequenced and phylogenetically compared to a comprehensive genome database of *A. thaliana* ecotypes. Experimental results on primary and secondary metabolite profiling and genotypic variation were functionally integrated by a data mining strategy, which combines statistical output of metabolomics data with genome-derived biochemical pathway reconstruction and metabolic modeling. Correlations of biochemical model predictions and population-specific genetic variation indicated varying strategies of metabolic regulation on a population level which enabled the direct comparison, differentiation, and prediction of metabolic adaptation of the same species to different habitats. These differences were most pronounced at organic and amino acid metabolism as well as at the interface of primary and secondary metabolism and allowed for the direct classification of population-specific metabolic phenotypes within geographically contiguous sampling sites.

## Introduction

Natural variation, as first described by Darwin (1859), is the ultimate point of attack for natural selection and still the only known process that is able to produce adaptive evolutionary change. *Arabidopsis thaliana* has become a powerful model organism for studying many aspects of plant biology and adaptation to the environment (Somerville and Koornneef, 2002; Hancock et al., 2011). After the publication of a first complete reference genome sequence (Arabidopsis, 2000), it was discovered that it is inappropriate to think about ‘the’ genome of a species (Weigel and Mott, 2009). In fact, all species are exposed to specific environmental clines differently affecting individual plants’ phenotypic performance (Turesson, 1922; Ellenberg, 1953; Hoffmann, 2002; Weckwerth, 2003, 2011a; Lasky et al., 2012; Weigel, 2012). Therefore, they comprise different populations colonizing different habitats. These habitats may impose differing directions of natural selection upon the coenospecies, and thus, together with genetic drift, lead to diverging allele frequencies and to an inhomogeneous genetic structure. This inhomogeneity is called natural genetic variation and potentially provides insights in genome evolution, population structure, and selective mechanisms (Mitchell-Olds and Schmitt, 2006). However, the genetic side represents only one level in the complex molecular architecture, which builds up the basis for physiological and morphological responses of plants to environmental stimuli (Pigliucci, 2010). The experimental analysis and interpretation of these molecular architectures is nonintuitive, particularly because of the highly complex organization of plant molecular networks. Numerous studies have shown that a multitude of genes, proteins, metabolites, and underlying regulatory processes are involved in plant-environment interactions (Koornneef et al., 2004; Wienkoop et al., 2008; Keurentjes, 2009; Chan et al., 2010; Macel et al., 2010; Lasky et al., 2012). However, interpreting these findings in the context of environmental conditions and, particularly, in an ecological context is highly challenging. This is particularly due to a missing stringent definition of the genotype–phenotype relationship, which can hardly be expected to be derivable from a single methodology but rather from a comprehensive platform of experimental and theoretical strategies (Weckwerth, 2003, 2011a; Diz et al., 2012). Recording environmentally induced fluctuations in a metabolic homeostasis has been shown to be a promising approach to unravel complex patterns of metabolic regulation and adaptation. For example, the metabolism of floral anthocyanins, which is a central group of secondary metabolites, was found to represent a suitable metabolic system to characterize the process of environmental regulation (Lu et al., 2009). The authors suggested that environmental regulation of the anthocyanin pathway is mainly affected by daily average temperature and UV light intensity modulating anthocyanin transcript levels at floral developmental stages. In another study, a metabolomics approach has been applied to elucidate *in situ* allelopathic relationships of individual species to phytosociological gradients (Scherling et al., 2010). We demonstrated that *in situ* metabolic signatures of five different plant species correlated with a biodiversity gradient. More general, metabolomics approaches can be expected to provide detailed information about metabolic processes in context of genomic signatures (Chae et al., 2014). Particularly in model systems with functionally annotated genomes this makes it the method of choice to unravel and interpret molecular ecological properties.

For the genetic and molecular biological model plant *A. thaliana*, one of the best functionally annotated genomes (Baerenfaller et al., 2012; Lavagi et al., 2012) and a comprehensive catalog of genome information is available^1^. Recently, an *in vitro* study of the physiological homeostasis of 92 *A. thaliana* accessions in multiple growth settings has demonstrated the devastating impact of varying environmental conditions on the correlation of *in vitro* metabolism to geographic origin (Kleessen et al., 2012). Yet, as microhabitats may vary significantly on relatively small spatial scales and are not necessarily corresponding to geographic distance, the investigation of the molecular performance of plants *in situ* seems inevitable to get a realistic picture of plant–environment interactions and their ecophysiological consequences. A well-known example indicating the need of such *in situ* studies is Ellenberg’s Hohenheimer groundwater table experiment (Ellenberg, 1953; Hector et al., 2012). Here, it was shown that the phenotypic performance of plants *in vitro* significantly differ from their *in situ* physiological homeostasis, as important microhabitat parameters may not be included in the *in vitro* growth setting (Shulaev et al., 2008). Both plant communities and plant populations seem to be an appropriate target for the development and tuning of *in situ* methodologies due to their sessile nature and the availability of a large set of *in vitro* reference data for some species. This enables the intersection of individual molecular with environmental data, and even ecosystem properties can be accounted for via geographic information systems. Genotyping approaches in *A. thaliana* have already been established (Atwell et al., 2010; Platt et al., 2010; Todesco et al., 2010; Hancock et al., 2011; Horton et al., 2012; Long et al., 2013) and are easily transferable to *in situ* samples (Hunter et al., 2013). Metabolomics and proteomics technologies provide the means for generating upstream molecular phenotypes (Morgenthal et al., 2005; Hoehenwarter et al., 2008; Wienkoop et al., 2008; Scherling et al., 2010; Weckwerth, 2011a; Doerfler et al., 2013). Thus, these techniques are suitable for experimental high-throughput analysis at the molecular level, representing the basis for strategies of multivariate statistics and mathematical modeling to identify biochemical perturbation sites and gain predictive power (Nägele and Weckwerth, 2013; Nägele, 2014). In this context, particularly metabolomic analysis has proven to be a suitable approach for the comprehensive and representative investigation of complex metabolic networks with respect to the underlying phenotypic diversity (Weckwerth et al., 2004a; Keurentjes, 2009; Scherling et al., 2010).

**FIGURE 1:**
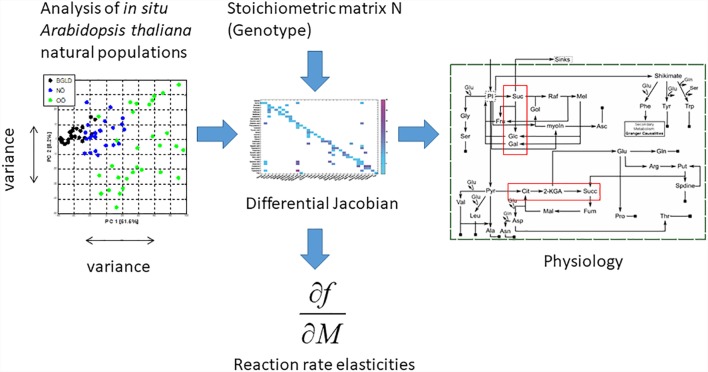
By the application of metabolomics, multivariate statistics and mathematical modeling based on genome-derived biochemical pathway information, biochemical and physiological signatures of *in situ Arabidopsis* populations can be identified. Different metabolic steady states on a population level and general patterns common to all populations can be distinguished by this metabolic modeling approach in combination with eco-metabolomics. This finally allows for the prediction of characteristic processes of *in situ* metabolic adaptation. For details see text and Materials and Methods.

In the present study, the genomes and metabolomes of *in situ* samples from three Austrian natural populations of *A. thaliana* were characterized. Applying a combination of metabolomics, multivariate statistics, and mathematical modeling based on genome-derived biochemical pathway information, biochemical and physiological signatures of *in situ Arabidopsis* populations could be identified (Figure 1). Different metabolic steady states on a population level and general patterns common to all populations were distinguished by this integrative approach, which finally allowed the prediction of characteristic processes of *in situ* metabolic adaptation.

## Materials and Methods

### Plant Material and Sampling Strategy

**FIGURE 2:**
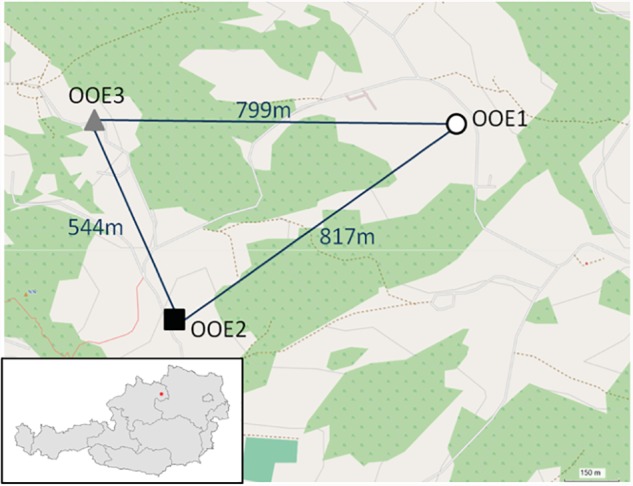
Projection of sample coordinates within OpenStreetMap^®^ (http://www.openstreetmap.org). The air-line distance between populations is given in meters.

*In situ* sampling of *A. thaliana* leaf rosettes was performed in three Austrian locations (see Figure 2) The first location (OOE1) was a hay meadow, the second (OOE2) was a rocky spot with variable substrate thickness, and the third sampling site (OOE3) was an unused meadow with steep slope and a nearby valley. All populations were located in close proximity to intensively used grassland. Each sample consisted of one whole leaf rosette without inflorescence. Global positioning system (GPS) coordinates of the sampling sites were recorded using a Garmin Oregon300 handheld GPS receiver (Garmin^®^, Schaffhausen, Switzerland) with an accuracy of approximately 3 m. The waypoints were imported into Garmin Mapsource Version 6.15.6 (Garmin^®^, Schaffhausen, Switzerland) and projected on the OpenStreetMap^2^. The sampling was performed according to Scherling et al. (2010) with a minimized cycle of time accounting for diurnal changes. The sampling began at 12 am at OOE1, then OOE2 and OOE3. The sampling was repeated three times at the same day comprising about 20 min each and was finished at 4 pm. The sampling day had continuous cloudy and constant weather conditions. All *Arabidopsis* rosettes were sampled at a developmental stage in which inflorescence and mature leaf rosettes had been established (example pictures are provided in Supplementary Data Sheet S1). Altogether we sampled *n* = 13, 15, and 15 biological replicates for OOE1, OOE2, and OOE3, respectively, for GC-MS and *n* = 10, 7, and 13 biological replicates for OOE1, OOE2, and OOE3, respectively, for LC-MS analyses. Rosettes were cut and immediately frozen in liquid nitrogen. Samples were stored at -80°C until further processing.

### DNA Sequencing and SNP Calling

Sequencing was performed for individual plants of the different populations OOE1, OOE2 and OOE3. Genomic DNA preparation, and SNP calling was performed as described previously (Alonso-Blanco et al., 2016). The samples were sequenced using 100 bp paired-end reads on an Illumina HiSeq platform. Pairwise genetic differences (θ_p_) between these accessions and a set of additional 24 accessions for which DNA sequence is publically available (see footnote 1) has been calculated by dividing the number of polymorphic sites by the number of informative sites. These values have been used to create a hierarchical clustering using the McQuitty method within the function *hclust* in R (McQuitty, 1966)^3^. To extract the most diverse genes from the three populations, we calculated the amount of variation between the populations for each gene. We used only sides where we had SNP calls for one representative of each population. We created a list for each population containing only genes that differ by at least 50 polymorphisms from the other two populations. These lists are available as Supplementary Data Sheets S2–S4. Furthermore, we created population-specific clustered protein interaction networks with these genes using STRING (Szklarczyk et al., 2017). In Supplementary Presentation S1 the networks and gene functions are shown. All SNP data are stored at the public repository (see footnote 1).

### Gas Chromatography Coupled to Time-of-Flight Mass Spectrometry

Frozen sample rosettes were homogenized in a ball mill (Retsch^®^, Haan, Germany) under frequent cooling with liquid nitrogen for 3 min. Polar metabolites were extracted and derivatized as described previously (Weckwerth et al., 2004b). Gas chromatography coupled to mass spectrometry (GC-MS) analysis was performed on an Agilent 6890 gas chromatograph (Agilent Technologies^®^, Santa Clara, CA, United States) coupled to a LECO Pegasus^®^ 4D GCxGC-TOF mass spectrometer (LECO Corporation, St. Joseph, MI, United States). Compounds were separated on an Agilent HP5MS column (length: 30 m length, diameter: 0.25 mm, film: 0.25 μm). Deconvolution of the total ion chromatograms was performed using the LECO Chromatof^®^ software. All details about injection, gradient, deconvolution, and library search parameters can be found in Doerfler et al. (2013). A calibration curve was recorded for absolute quantification of central primary metabolites.

### GC–MS Data Analysis and Inverse Approximation of Jacobian Matrix Entries

For ANOVA and computation of *p*-values adjusted for sample size by Tukey Honest Significant Differences R was used (R, 2013). For multivariate analysis, outliers (all values that were lower/higher than 1.5^∗^interquartile range from the 25%/75% quantile) were removed from the dataset. Missing values of variables, which were missing in more than half of all measurements in a population were filled with half of the matrix minimum. The remaining missing values were imputed by random forest computation (Stekhoven and Buhlmann, 2012; Gromski et al., 2014). This dataset was centered and scaled to unit variance prior to sPLS regression. Sparse partial least squares (sPLS) regression analysis was performed using the mixOmics package (Le Cao et al., 2009; Gonzalez et al., 2011, 2012) for the statistical software environment R (R, 2013).

The functional integration of GC–MS metabolomics data into a metabolic network was performed, as previously described (Nägele et al., 2014), by the approximation of the biochemical Jacobian matrix. This approximation directly connects the covariance matrix C, which was built from the experimental metabolomics data, with a metabolic network structure derived from *Arabidopsis* genome information. Linkage of covariance data with the network structure follows equation 1 (Steuer et al., 2003; Sun and Weckwerth, 2012):

(1) $$JC+{CJ}^{T}=-2D\,$$

Here, *J* represents the Jacobian matrix and *D* is a fluctuation matrix which integrates a Gaussian noise function simulating metabolic fluctuations around a steady state condition. In context of a metabolic network, entries of the Jacobian matrix *J* represent the elasticity of reaction rates to any change of metabolite concentrations which are characterized by equation 2:

(2) $$J=N\frac{\partial r}{\partial M}$$

*N* is the stoichiometric matrix or a metabolic interaction matrix if reactions and reactants have been modified in the original network. *r* represents the rates for each reaction, and *M* represents metabolite concentrations. As stated before, the Jacobian approximation comprises the stochastic term *D*. Therefore, we performed 10 × 10^5^ inverse approximations for each population, finally resulting in 10 technical replicates of the Jacobian matrices.

All calculations of Jacobian matrices were performed based on a modified version of the toolbox COVAIN (Sun and Weckwerth, 2012) within the numerical software environment MATLAB^®^ (V8.4.0 R2014b).

### LC–MS Analysis

The frozen plant leaf material was homogenized and extracted as the samples for the GC–MS analysis as described recently (Weckwerth et al., 2004b; Doerfler et al., 2013). The polar fraction of metabolites was dried in a speedvac. Extracts were weighed and dissolved in 5% Acetonitrile 0.5% Formic acid to an extract concentration of 0.5 g/L. From these solutions, 3 μL where injected to an Agilent Ultimate 3000 LC-system and separated on a reversed-phase column on a 60-min effective gradient prior to data-dependent mass spectrometric analysis of +1 – charged ions (Doerfler et al., 2013, 2014). Acquired LC–MS runs were converted to the open mzXML data format using the MassMatrix File Conversion Tools. Subsequently, MS1 intensities of all mass traces that were fragmented at least once in a sample were summed over the whole runs with ProtMAX2012 (Hoehenwarter et al., 2011; Doerfler et al., 2013; Egelhofer et al., 2013). The data set was filtered for features that were measured in at least half of the replicates of one population and remaining variables were normalized to the sum of all variables of the respective sample. The resulting values were used to fit ANOVA models. Tukey Honest Significant Differences were used to estimate sample-size adjusted *p*-values in R (R, 2013). VENNY was used to visualize the number of detected significant differences (Oliveros, 2007).

For multivariate analysis, outliers (all values that were lower/higher than 1.5^∗^interquartile range from the 25%/75% quantile) were removed from the dataset. Missing values of variables, which were missing in more than half of all measurements in a population were filled with half of the matrix minimum. The remaining missing values were imputed by random forest computation (Stekhoven and Buhlmann, 2012; Gromski et al., 2014). This dataset was centered and scaled to unit variance prior to sPLS regression (see above).

## Results

### Metabolomic Analysis of *in situ* Samples

**FIGURE 3:**
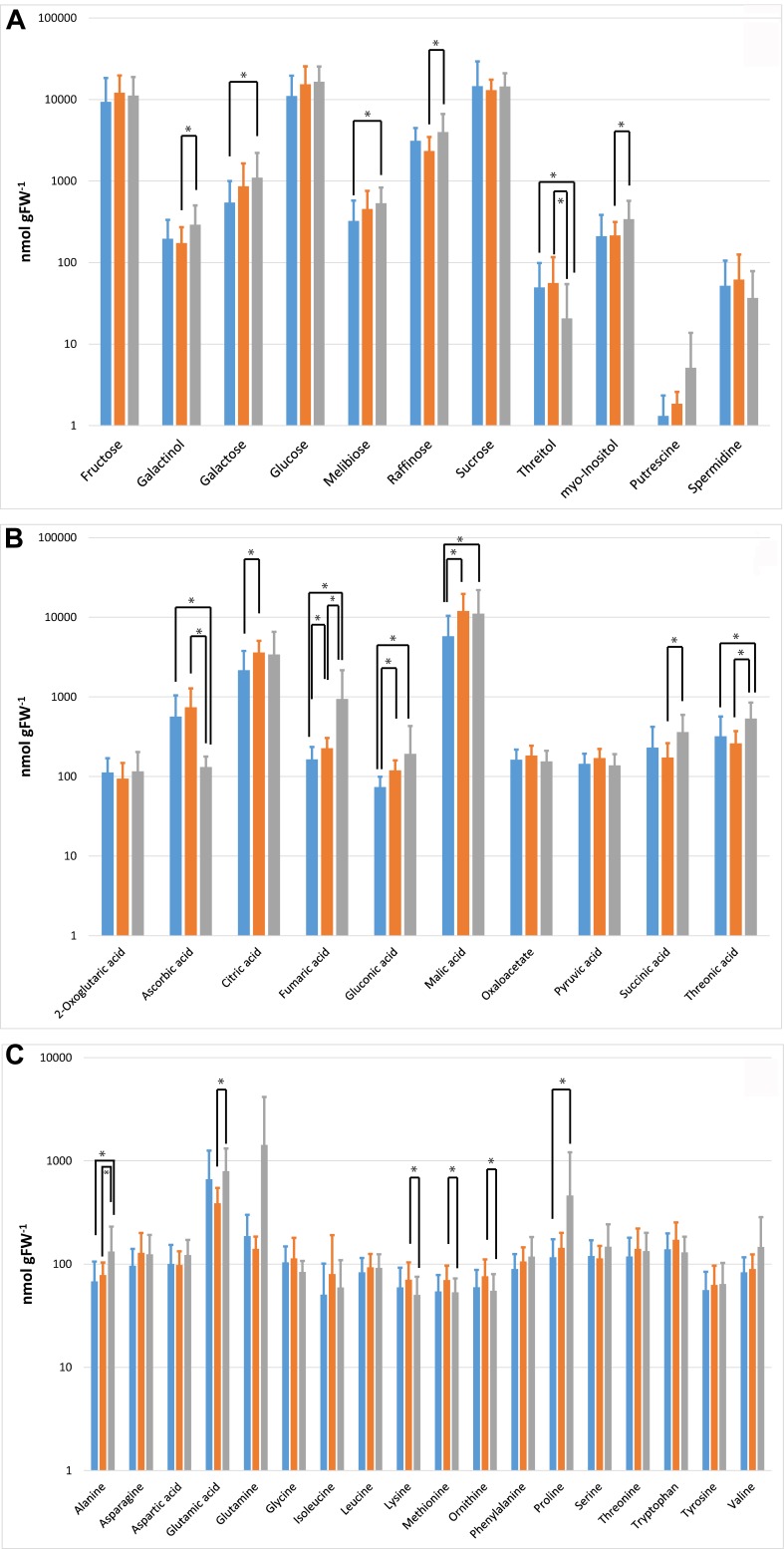
Absolute levels of primary metabolites. Metabolites are grouped according to the substance classes of **(A)** sugars, sugar alcohols, and polyamines; **(B)** organic acids; **(C)** amino acids. Significant differences evaluated by ANOVA are indicated by asterisks (^∗^*p* < 0.05). Metabolite levels from samples of OOE1 are indicated by blue bars, OOE2 by orange bars, and OOE3 by gray bars.

*In situ* sampling of *A. thaliana* leaf rosettes was performed on three nearby locations in Upper Austria (Oberoesterreich; OOE; see Figure 2 and See section “Materials and Methods”). All *Arabidopsis* rosettes were sampled at a developmental stage in which inflorescence and mature leaf rosettes had been established (example pictures are provided in Supplementary Data Sheet S1). For a set of metabolites from untargeted GC–MS based metabolomics data, we performed absolute quantification using calibration curves. This set of metabolites comprised concentrations of 39 central compounds of the C/N metabolism including sugars and sugar alcohols, organic acids, amino acids, and polyamines (Figure 3). Results of an ANOVA indicated that only levels of fumaric acid discriminated all three populations significantly (Figure 3B). Populations OOE1 and OOE3 could be discriminated significantly by the concentrations of galactose, melibiose, threitol, ascorbic acid, fumaric acid, gluconic acid, malic acid, threonic acid, alanine, and proline (*p* < 0.05; Figure 3). For populations OOE2 and OOE3, significant differences were found to exist for absolute levels of galactinol, raffinose, threitol, myo-inositol, ascorbic acid, fumaric acid, succinic, and threonic acid as well as for the amino acids alanine, glutamic acid, lysine, methionine, and ornithine (*p* < 0.05; Figure 3). Populations OOE1 and OOE2 could be discriminated by levels of citric acid, fumaric acid, gluconic acid, and malic acid. To summarize these findings, most significant differences between absolute metabolite levels of populations OOE1, 2, and 3 were determined for the class of organic acids (13 out of 27, i.e., ∼50%).

### Multivariate Analysis Indicates a Discrimination of *in situ* Populations by Metabolic Phenotypes

**FIGURE 4:**
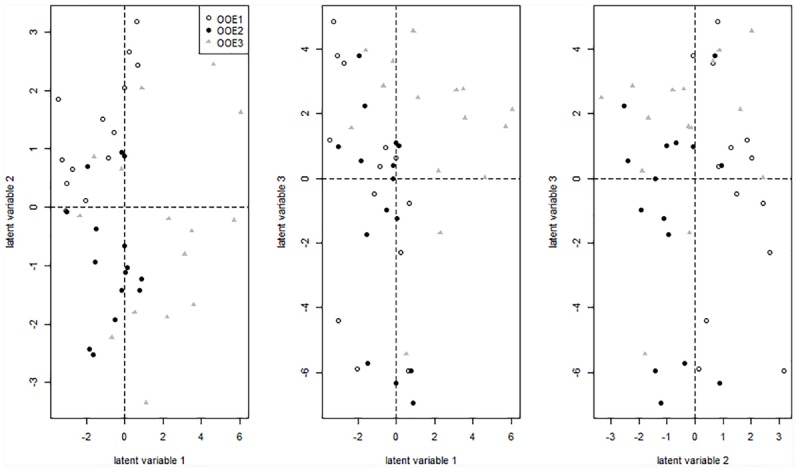
Projection of samples on latent variables of the primary metabolite matrix (GC-MS data) after sPLS regression. Detailed information on the loadings are provided in the supplement (Supplementary Table S1).

Sparse partial least squares (sPLS) regression analysis of primary metabolites versus a response matrix comprising geographical coordinates and altitude above sea level indicated a separation of population OOE3 from populations OOE1 and OOE2 across latent variable 1 (Figure 4). The metabolite levels of fumaric acid, melibiose, alanine, putrescine, gluconic acid, threonic acid, myo-inositol, galactinol and succinic acid were identified to contribute most to this separation with elevated levels in OOE3 whereas mainly ascorbic acid and threitol were elevated in OOE1 and OOE2. Discrimination of populations OOE1 and OOE2 was indicated on latent variable 2 (Figure 4). Here, a higher abundance of 2-oxoglutaric acid, glutamic acid, raffinose, glycine, succinic acid, serine and threonic acid in OOE1 and malic acid, gluconic acid and citric acid in OOE2 was observed (see Supplementary Table S1 for a complete list of loadings, table sheet “Loadings GCMS”, and Supplementary Figure S1 for a PCA analysis of the primary metabolites).

### Entries of Jacobian Matrices Indicate Different Biochemical Phenotypes of the *in situ* Populations at the Interface of Primary and Secondary Metabolism

**FIGURE 5:**
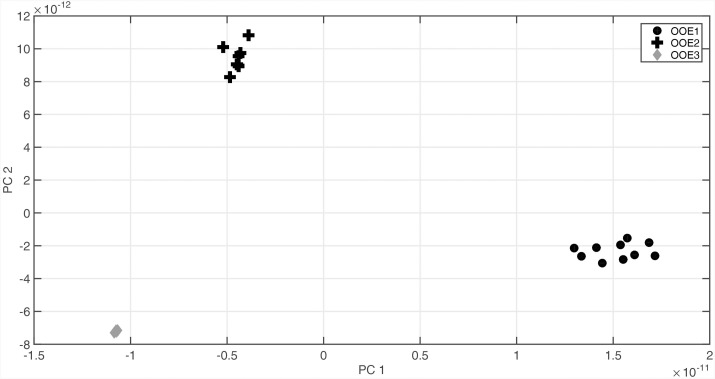
Principal component analysis (PCA) of Jacobian matrix entries for populations OOE1, OOE2, and OOE3. PC1 strongly separates OOE1 (black filled circles) from OOE2 (black filled crosses) and OOE3 (gray filled diamonds). PC2 separates OOE2 from OOE1 and OOE3 most significantly. Detailed information about the Jacobian entries and the loadings can be found in Supplementary Tables S2, S3.

**FIGURE 6:**
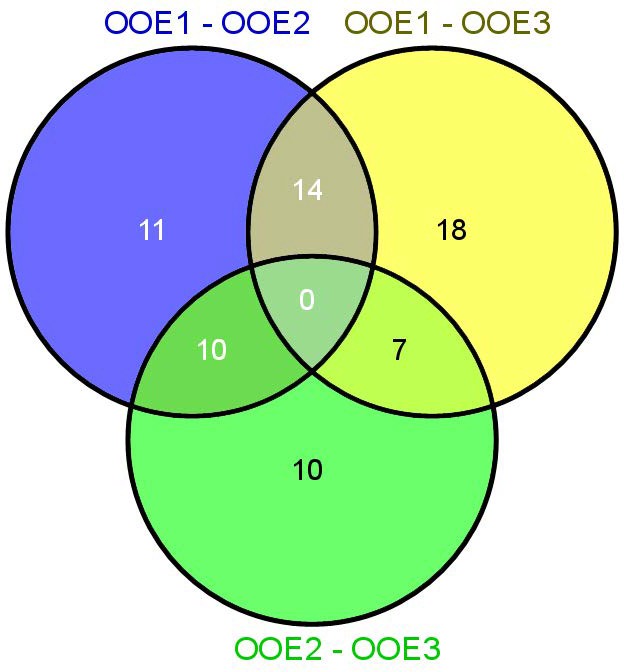
Venn diagram showing the number of LC–MS features, which significantly discriminated the three *Arabidopsis* populations OOE1, 2, and 3.

While absolute metabolite levels can provide a representative view on a metabolic homeostasis, it can hardly be directly interpreted in terms of biochemical regulation. Instead, strategies of multivariate statistics and modeling were shown to be essential to provide a comprehensive view on the biochemical regulation of a metabolic homeostasis (Weckwerth, 2011b). Based on a biomathematical strategy developed and applied in former studies, entries of Jacobian matrices were directly inferred from experimental metabolomic covariance data (Doerfler et al., 2013; Nägele et al., 2014) (Figure 1). As described in our previous work, we derived a metabolic network model comprising reactants and reactions indicated in the Supplementary Table S2. The metabolic covariance information was linked to a genome-information derived biochemical network structure, finally satisfying a Lyapunov matrix equation [for more details about the method and the metabolic network model, we refer to the section Materials and Methods as well as to our previous work (Nägele et al., 2014)]. The calculation procedure, that is, solving the equation after stochastic perturbation, was performed 10 × 10^5^ times and median values of all entries of the Jacobian matrices were determined. Principal component analysis (PCA) of the entries revealed a clear separation of the population-specific Jacobian information in which the technical variance was found to be significantly lower than the biological variance (Figure 5). Loadings of the PCA revealed that the strong separation of population OOE1 from OOE2 and 3 on component 1 (PC1) was predominantly due to differences in organic and amino acid, polyamine, and raffinose metabolism but also aromatic amino acid biosynthesis and interconversion (Supplementary Tables S2, S3). This output indicated a potential difference in the regulation of secondary metabolism, or, at least, at the interface between primary and secondary metabolism. Hence, secondary metabolite abundance of the three Austrian *Arabidopsis* populations was recorded applying LC–MS analysis. The quantitative analysis of specific mass traces in the chromatograms showed that there was no feature separating all of the populations significantly (ANOVA, *p* < 0.05). Yet, we were able to identify 70 features that discriminated at least two of the populations (Figure 6).

**FIGURE 7:**
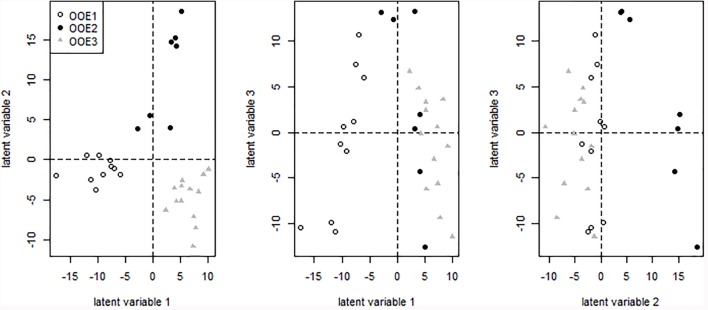
Projection of samples on latent variables of the secondary metabolite matric (LC–MS data) after sPLS regression. Detailed information on the loadings are provided in the supplement (Supplementary Table S1).

To statistically evaluate the separation of populations by secondary metabolites, LC–MS data were analyzed by sPLS regression analysis. The first latent variable was found to separate OOE1 from OOE2 and OOE3 (Figure 7; Loadings are provided in Supplementary Table S1, table sheet “Loadings LCMS”). The second latent variable indicated a separating effect of several putative anthocyanins attached to sinapoyl moieties [A6, A7/A17, A8, A10, A11, and *m/z* 1329, respectively, for further annotation see Doerfler et al. (2014)] in the OOE2 population by which it was discriminated from OOE1 and OOE3.

### Genotyping of *in situ* Natural *A. thaliana* Populations

**FIGURE 8:**
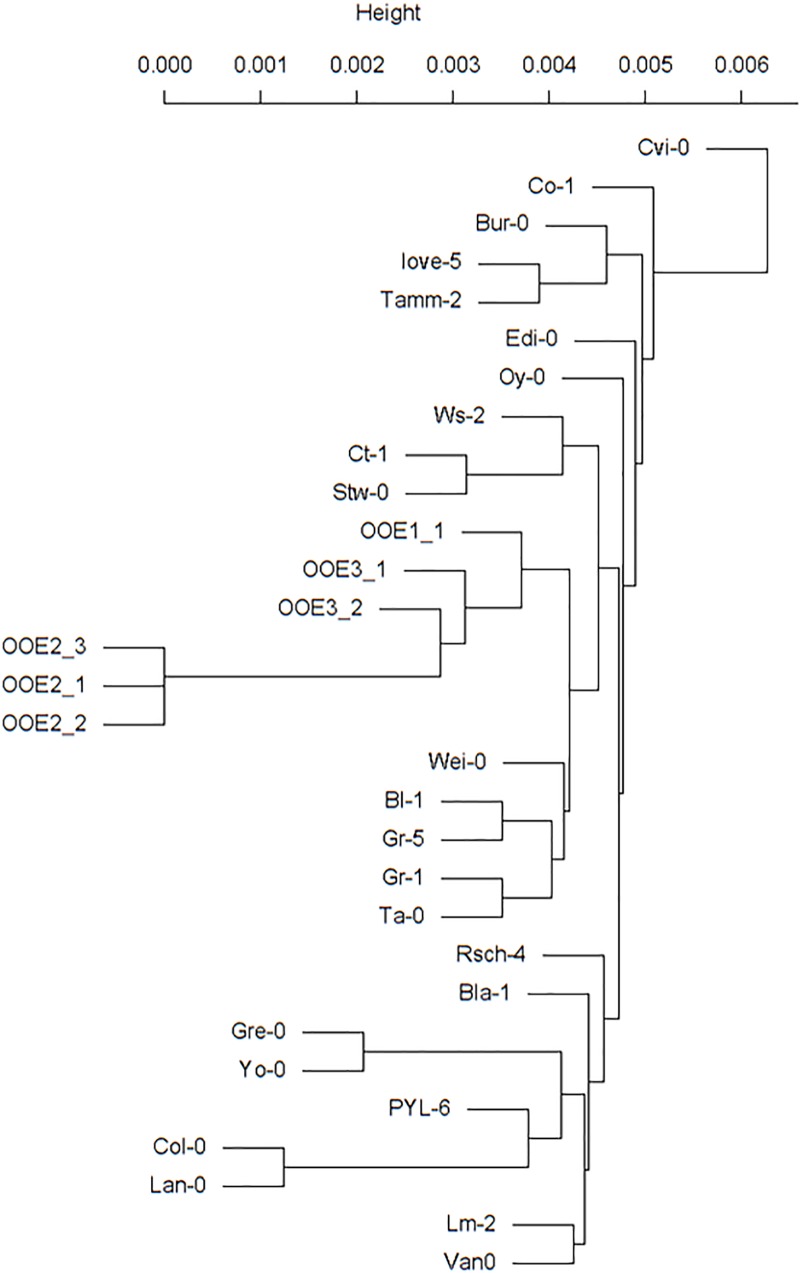
SNP genotyping of three Austrian *Arabidopsis* populations. All three plants that have been sequenced from the population OOE2 are nearly genetically identical. OOE2 differs by nearly 300,000 SNPs from both the OOE1 and OOE3 population, which are likewise separated by more than 300,000 SNPs. The comparison with genomic data from other ecotypes showed the expected genetic differences not only within these populations but also to global samples, in which accessions from Austria, Italy, and the Czech Republic are most related. The genome information of all accessions is publically available at www.1001genomes.org.

A SNP-based genotyping approach was performed to unravel the genomic relationship of the three populations. Genotyping showed clear differences between the three populations (Figure 8). Different individual plants of population OOE2 were found to be nearly identical (12, 23, and 13 SNPs, respectively). The population OOE2 was found to differ by approximately 300,000 SNPs from both populations OOE1 and OOE3, which were likewise separated by more than 300,000 SNPs. Interestingly, individual plants that have been sequenced from the OOE3 population were genetically different as well but to a minor extent (∼260,000 SNPs). The comparison with genomic data from other ecotypes show the expected genetic differences not only between these populations but also with respect to global samples, in which accessions from Austria, Italy, and the Czech Republic are most similar (Figure 8). To extract the most diverse genes from the three populations, we created a list for each population containing only genes that differ by at least 50 polymorphisms from the other two populations. These lists are available as Supplementary Data Sheets S2–S4. Furthermore, we created population-specific clustered protein interaction networks with these genes using STRING (Szklarczyk et al., 2017). These protein interaction networks showed highly diverse functional pattern between the three different populations (see Supplementary Presentation S1).

## Discussion

### Eco-Metabolomics and Metabolic Modeling

The importance and central role of metabolomics in an ecological context has extensively been outlined in previous studies and overview articles [see e.g., Sardans et al., 2011; Jones et al., 2013). One of the central issues of eco-metabolomic approaches is the detection and characterization of environmentally induced phenotypic mechanisms in context of key metabolic processes and ecologically relevant parameters, that is, all kinds of environmental cues (Scherling et al., 2010). Yet, due to the non-linear relationship between single levels of molecular organization, the reliable interpretation of metabolomics results is highly challenging. The metabolic output or homeostasis of a biochemical system depends on numerous molecular parameters and variables, and, finally, a metabolic network sums up to a highly branched, interlaced and non-linearly behaving molecular system (Nägele, 2014).

While under controlled conditions such plasticity of molecular systems already significantly limits our ability to intuitively draw conclusions about regulatory mechanisms, *in situ* data interpretation has to cope even more with a potential ambiguousness introduced by environmental dynamics and fluctuations (Macel et al., 2010). In the present study, such fluctuations were taken into account by considering (co)variance information of metabolite pools and by a modeling approach, which focuses on the characterization of dynamical behavior of metabolic systems around a metabolic homeostasis (Nägele et al., 2014) (see Figure 1). In detail, data dimensionality reduction via PCA indicated a clear separation of all populations by Jacobian entries being related to the biochemistry at the interface of primary and secondary metabolism as well as the metabolism of metabolic stress-markers, such as polyamines and raffinose, which have been discussed to be involved in the protection of the photosynthetic apparatus against various stress types (Bouchereau et al., 1999; Alcázar et al., 2006, 2011; Knaupp et al., 2011).

### Plasticity of Plant Primary Metabolism in *in situ* Populations and Correlation With Geographical Coordinates

Statistics on absolute primary metabolite levels revealed major differences between natural *in situ* Austrian *A. thaliana* populations. Almost all classes of analyzed substances, comprising sugars, carboxylic, and amino acids displayed significant differences indicating different homeostasis in primary metabolism of all three populations. The TCA intermediate fumaric acid was found to significantly differ between all *in situ* samples indicating suitability to classify these populations. While it has been shown that fumaric acid metabolism plays a central role in diurnal carbon allocation (Pracharoenwattana et al., 2010), and, hence, indirectly affects the orchestration of photosynthesis in *Arabidopsis* leaves, it remains to be demonstrated whether it can directly report on changes in plant–environment interactions. In addition, due to the complex regulation of plant primary metabolism, it can hardly be assumed that one metabolite level provides representative information for robust metabolic *in situ* classification. Yet, together with the finding of a significant difference in potential photosystem-protective substances, for example, polyamines and flavonoids, it can be hypothesized that differential metabolic homeostasis evolved due to differences in the microenvironment of the three populations being characteristic enough to separate them according to the resulting metabolic signatures. We further asked the question whether we can identify metabolite marker, which show significant correlations with geographical coordinates even within this proximate distribution of populations. In Supplementary Presentation S2 a correlation network is shown between geographical coordinates and primary metabolites of the three different populations. Indeed, there is a clear distinction between several metabolites showing significant correlations to altitude, E and N coordinates. This further provides evidence that metabolic homeostasis is related to environmental differences between these different locations of the natural populations.

### The Interface of Primary and Secondary Metabolism as a Key Regulatory Point for Genotypic and Phenotypic Plasticity

Predictions about the differentiation via signatures in secondary metabolism were validated by LC–MS metabolomics focusing on a central set of secondary metabolite backbones with close similarity to previously identified anthocyanins attached to sinapoyl moieties (Doerfler et al., 2014). Such metabolic differences are in line with previous findings reporting on metabolic signatures, which are due to characteristic differences in specialized or secondary metabolism (Wink, 2003; Lu et al., 2009; Scherling et al., 2010; Doerfler et al., 2013; Chae et al., 2014; Moore et al., 2014). The accumulation of anthocyanin pigments in vegetative tissue of plants represents an approved metabolic stress and acclimation output (Winkel-Shirley, 2002). Moreover, we demonstrated earlier that most of the statistical significant metabolic responses of individual plant species to *in situ* biodiversity are attributed to secondary metabolism including flavonoid structures (Scherling et al., 2010). Hence, the molecular analysis provided a detailed view on the differential population-specific metabolic composition of secondary metabolites and anthocyanin-related leaf color. With this, evidence is provided for the suitability of metabolic phenotyping of *in situ* samples by a combined GC–MS and LC–MS platform (Scherling et al., 2010; Doerfler et al., 2013). While, at this point, we can only speculate on the environmental cues which initiated the observed differences in secondary stress-associated metabolism, flavonoid metabolites in general are heavily discussed in context of their UV absorption and reactive oxygen species (ROS) scavenging properties (Winkel-Shirley, 2002; Agati and Tattini, 2010; Doerfler et al., 2014; Hectors et al., 2014). Together with the finding of a differentially regulated polyamine metabolism between the populations, which became visible rather by covariance information than by mean values, our results point toward a differential macro- or microclimatic environment at the three Austrian *in situ* sampling sites (see also description of the sampling sites in Materials and Methods).

In addition, results of SNP-based genotyping revealed three genetically different populations, which are, however, closer related to each other than to other European accessions (Figure 8). In terms of temperature regimes and low temperature tolerance, which can be expected to have major influence on the geographic range of *A. thaliana* (Hoffmann, 2002), the genetic distance between the Austrian populations can be regarded as relatively small when compared to sensitive (Cvi, Co-1) and tolerant accessions (Rsch-4) (Hannah et al., 2006). Based on this observation, we hypothesize that the variance in observed metabolic phenotypes are a mixture of plasticity effects and conceptual differences in the acquisition of abiotic stress tolerance. This again might indicate a high intraspecific metabolic variation, which would affect the evolutionary capacity of *Arabidopsis* in context of adaptation to macro- and micro-environmental fluctuation (Moore et al., 2014).

### Jacobian Entries Are Potentially Linked to Intraspecific Genotypic Variation

The combination of in depth genotypic and metabolomic profiling and modeling opens up the opportunity to search for direct correlations of polymorphisms and metabolic changes. Here, we applied this concept to an *in situ* study for the first time and revealed a significant intraspecific biochemical plasticity within three close-by natural populations in their natural habitat. We have extracted the genes of the individual populations which distinguish them most (Supplementary Data Sheets S2–S4). By further analysis of the corresponding clustered protein interaction networks different functional modules between the different populations became visible (Supplementary Presentation S1). The three populations OOE1, 2, and 3 showed severe differences in these protein interaction networks. Especially, the OOE3 population showed a cluster of genes which is clearly involved in organic acid and amino acid metabolism including genes for pyruvate dehydrogenase, aconitase, NAD-malic enzyme 1, pyruvate–phosphate dikinase, lactate-dehydrogenase, and several others (see Supplementary Presentation S1). These functional patterns, which distinguish OOE3 from OOE1 and 2 coincide with the calculation of Jacobian entries. The strongest loadings separating OOE3, 2, and 1 on PC1 in Figure 4 include df(Glu)/d(Pyr), df(Mal)/d(Fum), df(Cit)/d(Pyr), df(Glu)/d(Asp), df(Glu)/d(2-oxoglutarate), df(Succ)/d(Put). All of these entries point to organic acid metabolism and key entry points for amino acid metabolism, especially nitrogen assimilation and transamination reactions. In future studies, we will investigate these relationships in more detail also by integrating proteomics studies. There is a great potential that the calculation of Jacobian entries of a biochemical matrix gives important clues about different dynamics in the same set of metabolites based on intraspecific but also interspecific genetic variance and biochemical regulation. This is due to the explicit linkage of the metabolite covariance matrix C – representing the dynamic part of the equation – and the Jacobian J, which relies on the metabolite interaction matrix defined by genome-scale metabolic reconstruction and biochemical pathways. Accordingly, the covariance matrix C is representative for the different ecotype dynamics whereas the Jacobian structure preserves the reconstructed metabolic network. Just the combination of both J and C in the Lyapunov matrix equation will reveal the dynamics of each ecotype individually (for further details see also Weckwerth, 2011a,b).

## Conclusion

In summary, it was demonstrated that intraspecific metabolic phenotypes of geographically nearby-grown *Arabidopsis* plants can be characterized and differentiated by their primary–secondary metabolic signature. In future studies, monitoring of micro-climatic properties will enable the characterization of sampling sites by continuous quantitative environmental data and thus improve the understanding of the ecological context of *in situ* molecular profiles. Additionally, biotic and abiotic habitat parameters, such as soil properties and phytosociological association, might even promote our current understanding of individual plants’ physiology. Finally, our study points to the importance of considering variance and covariance information in biological data sets (Weckwerth et al., 2004b; Violle et al., 2012) which, together with genome-derived pathway information, potentially provide information about environmental fluctuations, and associated biochemical system properties. The findings contribute to the comprehensive understanding of ecological processes and may contribute to the design of future studies focusing on the estimation of the impact of climate change on plant societies and evolution using combined multiomics and modeling strategies (Ward and Kelly, 2004; Weckwerth, 2011a).

## Author Contributions

MNa collected natural populations of *Arabidopsis thaliana*, performed the measurements and statistical analysis, and wrote the manuscript. TN performed the measurements, statistical analysis, metabolic modeling, and wrote the manuscript. CG identified and collected natural populations of *Arabidopsis thaliana*. LF harvested sample material. AK, AP, AF, and MNo performed SNP calling, population analysis, and wrote the manuscript. WW conceived the study, performed statistical analysis, and wrote the manuscript.

## Conflict of Interest Statement

The authors declare that the research was conducted in the absence of any commercial or financial relationships that could be construed as a potential conflict of interest.

## Footnotes

1 http://www.1001genomes.org

2 http://www.freizeitkarte-osm.de/de/oesterreich.html

3 https://www.rdocumentation.org/packages/stats/versions/3.5.1/topics/hclust

## Supplementary Material

Figure S1 PCA analysis of primary metabolites.

Table S1 PCA loadings of GC-MS and LC-MS metabolites.

Table S2 Table of Jacobian entries and their associated metabolite, pathway and enzyme reaction (EC number).

Table S3 PCA loadings of the Jacobien entries.

DATA SHEET S1 Examples of individual plants of the three *Arabidopsis thaliana* populations OOE1-3.

DATA SHEET S2 SNP enriched genes in OOE1.

DATA SHEET S3 SNP enriched genes in OOE2.

DATA SHEET S4 SNP enriched genes in OOE3.

PRESENTATION S1 String protein interaction networks for SNP enriched genes distinguishing the three natural *Arabidopsis thaliana* populations OOE1-3.

PRESENTATION S2 Metabolite – GPS – correlation network.
