# Supplementary figures and images for: Eco-Metabolomics and Metabolic Modeling: Making the Leap From Model Systems in the Lab to Native Populations in the Field
Source: Front Plant Sci. 2018 Nov 6;9:1556. doi: 10.3389/fpls.2018.01556 (PMC6232504; doi:10.3389/fpls.2018.01556)

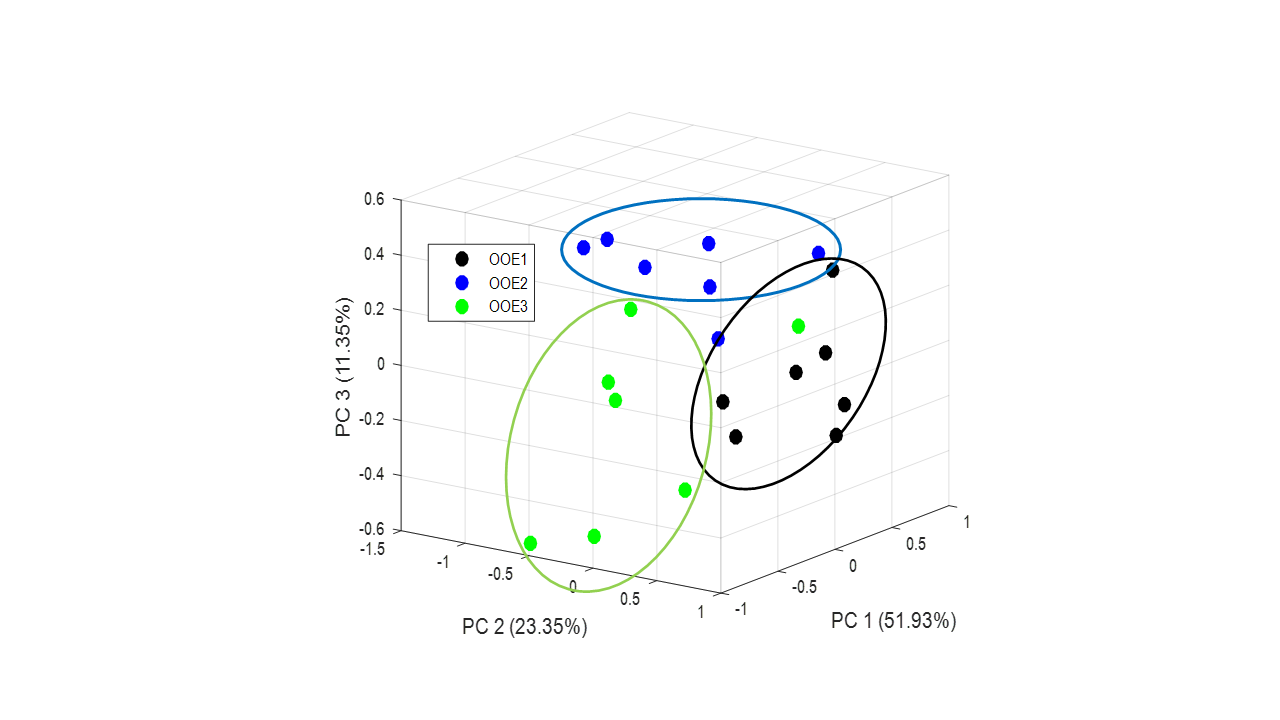

Supplement: Figure S1 — PCA analysis of primary metabolites. [file Image_1.TIF]

Sample of population OOE1

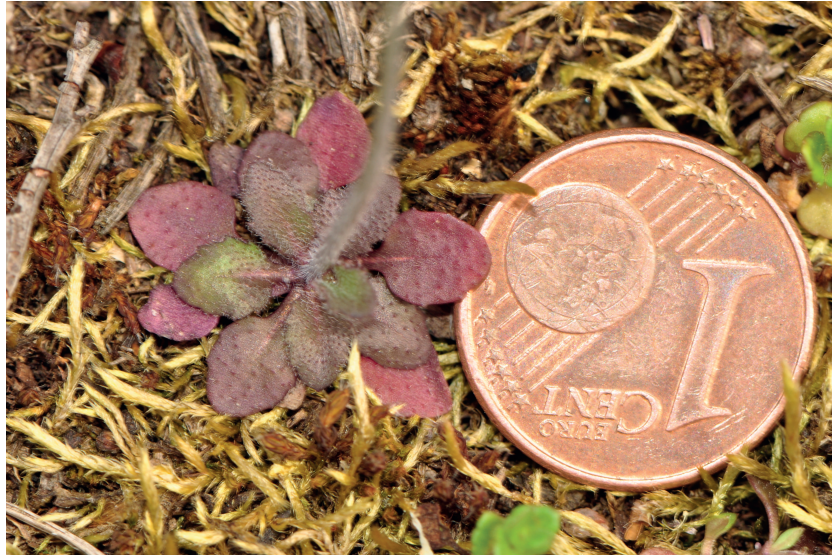

Sample of population OOE2

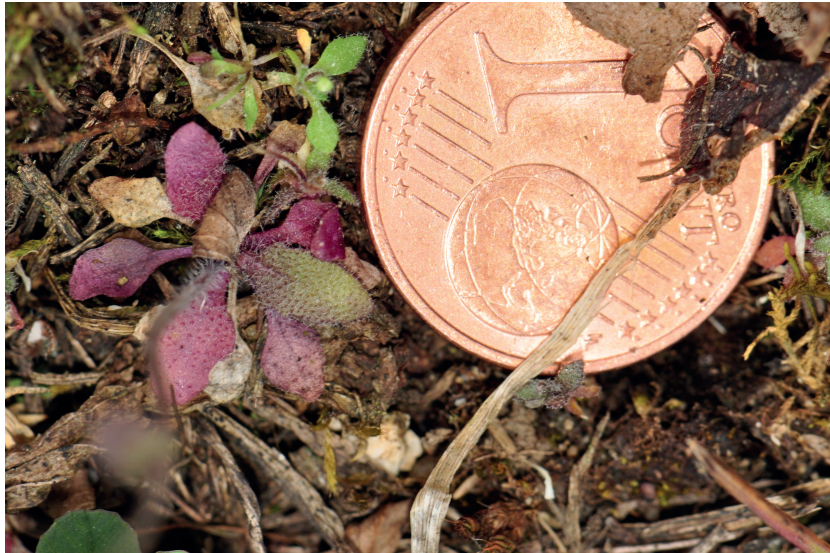

Sample of population OOE3

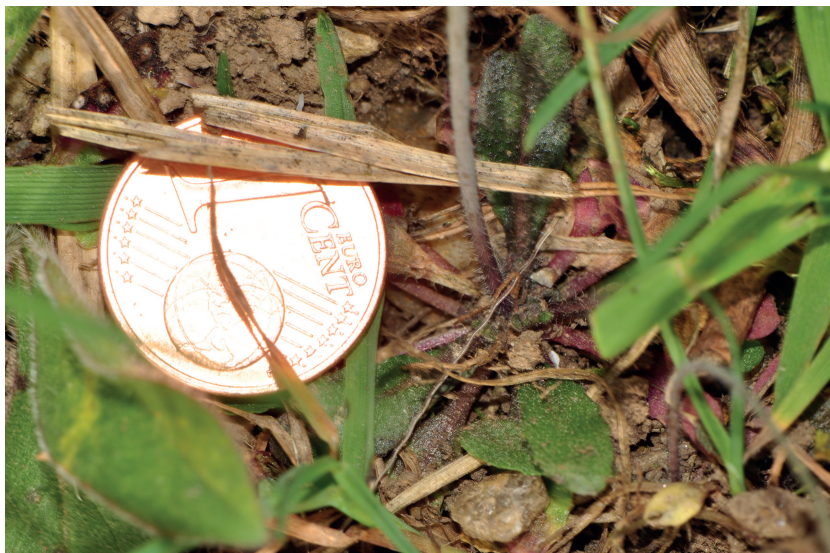

Supplement: DATA SHEET S1 — Examples of individual plants of the three Arabidopsis thaliana populations OOE1-3. [file Data_Sheet_1.PDF]

## Slide 1
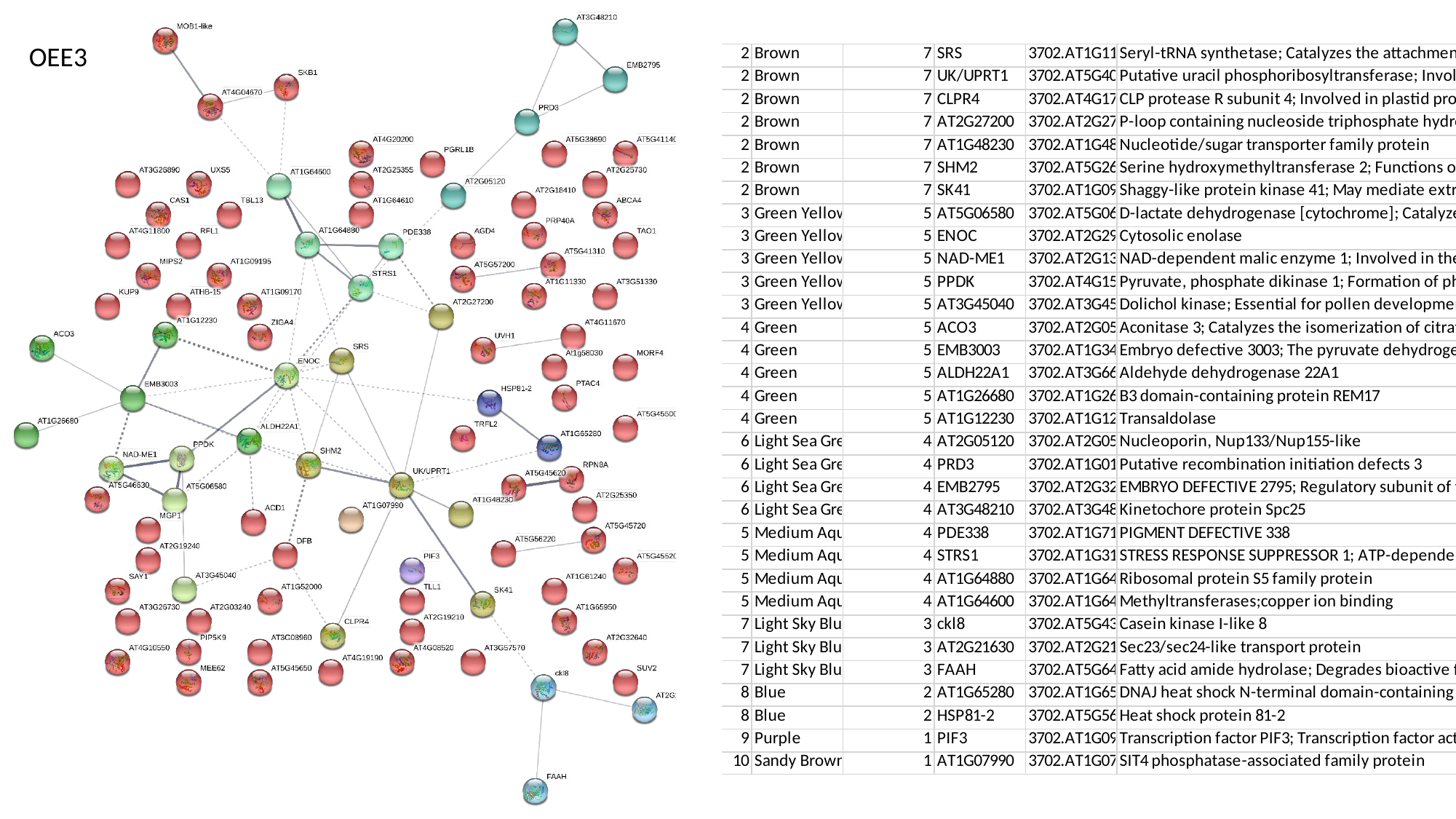

OEE3

## Slide 2
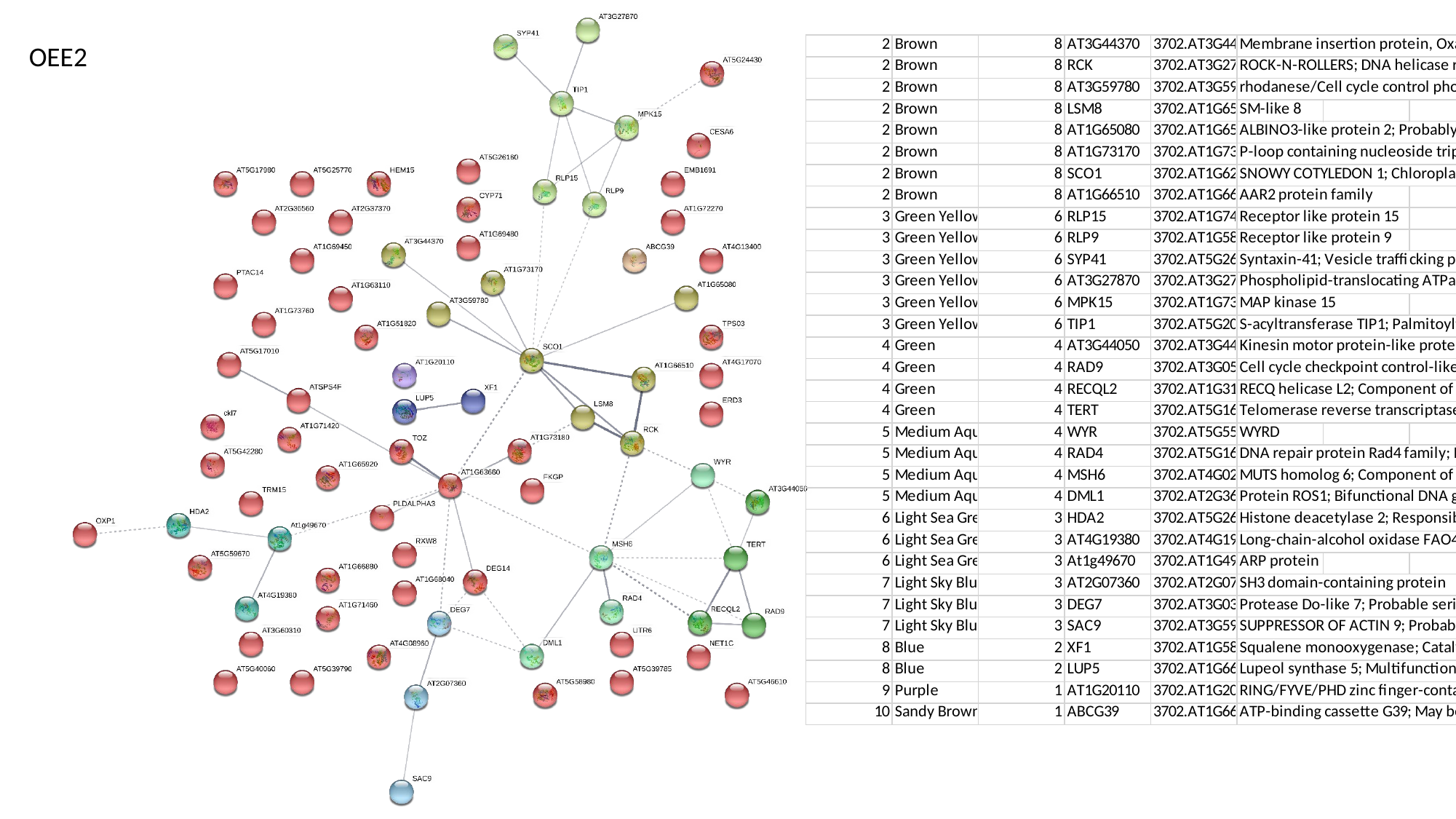

OEE2

## Slide 3
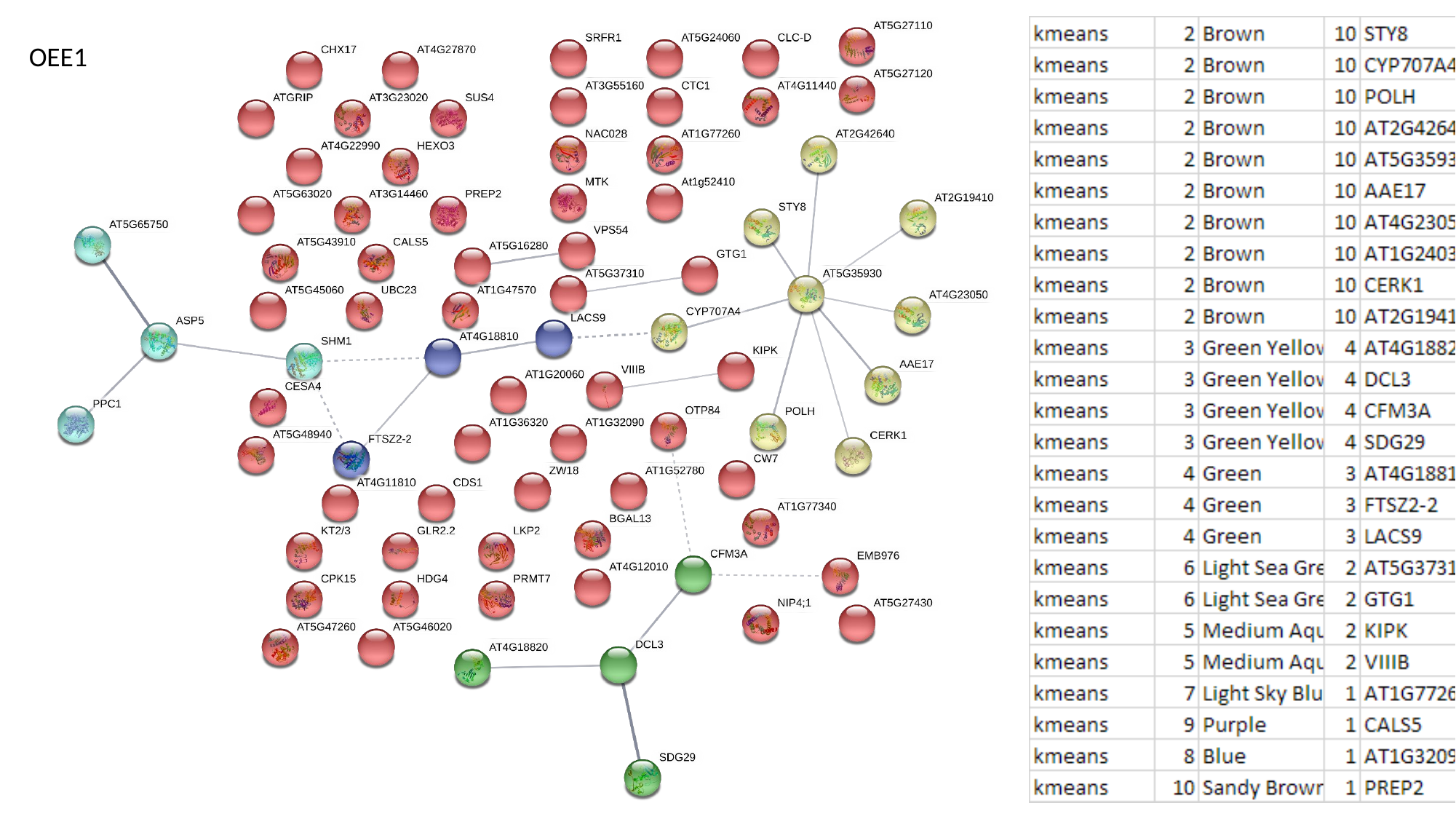

OEE1

Supplement: PRESENTATION S1 — String protein interaction networks for SNP enriched genes distinguishing the three natural Arabidopsis thaliana populations OOE1-3. [file Presentation_1.PPTX]
