# Supplementary material for: Eco-Metabolomics and Metabolic Modeling: Making the Leap From Model Systems in the Lab to Native Populations in the Field
Source: Front Plant Sci. 2018 Nov 6;9:1556. doi: 10.3389/fpls.2018.01556 (PMC6232504; doi:10.3389/fpls.2018.01556)
Supplement: Table S2 — Table of Jacobian entries and their associated metabolite, pathway and enzyme reaction (EC number). [file Table_2.DOCX]

| **Jacobian Entry**  **df[..]/d[..]** | | | **Associated Pathway** | **Representative**  **Enzyme Reaction**  **(EC Number)** |
| --- | --- | --- | --- | --- |
| **Function of**  **(df[..])** | **Variable**  **(d[…])** | |  |  |
| Glycine | Glycine | | Photorespiration | 2.1.2.1 |
|  | Glutamic acid | | Photorespiration | 2.6.1.4 |
| Serine | Glycine | | Photorespiration | 2.1.2.1 |
|  | Serine | | Photorespiration | 2.6.1.45 |
|  | Glutamine | | Tryptophan biosynthesis | 4.1.3.27 |
| Sucrose | Sucrose | | Sucrose export/interconversion/  cleavage | 2.4.1.82  3.2.1.26 |
|  | Galactinol | | Raffinose biosynthesis | 2.4.1.82 |
| Fructose | Sucrose | | Sucrose cleavage | 3.2.1.26 |
|  | Fructose | | Fructose interconversion/  phosphorylation | 2.7.1.4 |
|  | Raffinose | | Raffinose cleavage | 3.2.1.26 |
| Glucose | Sucrose | | Sucrose cleavage | 3.2.1.26 |
|  | Glucose | | Glucose interconversion/  phosphorylation | 2.7.1.1 |
|  | Melibiose | | Melibiose cleavage | 3.2.1.22 |
| Raffinose | Sucrose | | Raffinose biosynthesis | 2.4.1.82 |
|  | Raffinose | | Raffinose interconversion/cleavage | 3.2.1.26 |
|  | Galactinol | | Raffinose biosynthesis | 2.4.1.82 |
| Melibiose | Raffinose | | Raffinose cleavage | 3.2.1.26 |
|  | Melibiose | | Melibiose interconversion/cleavage | 3.2.1.22 |
| Galactinol | Sucrose | | Raffinose biosynthesis | 2.4.1.82 |
|  | Galactinol | | Raffinose biosynthesis | 2.4.1.82 |
|  | myo-Inositol | | Galactinol synthesis | 2.4.1.123 |
| Galactose | Galactose | | Galactose interconversion/  phosphorylation | 2.7.1.6 |
|  | Melibiose | | Melibiose interconversion/degradation | 3.2.1.22 |
| myo-Inositol | myo-Inositol | | myo-Inositol interconversion/  Galactinol synthesis | 2.4.1.123 |
| Phenylalanine | Phenylalanine | | Phenylalanine interconversion/degradation | 2.6.1.57  2.6.1.58 |
|  | Glutamic acid | | Phenylalanine biosynthesis | 2.6.1.79  4.2.1.91 |
| Tyrosine | Tyrosine | | Tyrosine interconversion/degradation | 2.6.1.5 |
|  | Glutamic acid | | Tyrosine biosynthesis | 2.6.1.79  1.3.1.78 |
| Tryptophan | Serine | | Tryptophan biosynthesis | 4.2.1.122 |
|  | Tryptophan | | Tryptophan interconversion/degradation | 2.6.1.27 |
|  | Glutamine | | Tryptophan biosynthesis | 4.1.3.27 |
| Pyruvic acid | Serine | | Tryptophan biosynthesis | 4.1.3.27 |
|  | Valine | | Alanine biosynthesis | 2.6.1.66 |
|  | Pyruvic acid | | Pyruvic acid interconversion/degradation | 1.2.4.1  2.6.1.58 |
|  | Glutamic acid | | Valine/Leucine/Alanine biosynthesis | 2.6.1.42  2.6.1.2 |
|  | Glutamine | | Tryptophan biosynthesis | 4.1.3.27 |
| **Jacobian Entry**  **df[..]/d[..]** | | | **Associated Pathway** | **Representative**  **Enzyme Reaction**  **(EC Number)** |
| **Function of**  **(df[..])** | | **Variable**  **(d[…])** |  |  |
| Valine | | Pyruvic acid | Valine biosynthesis | 2.6.1.42 |
|  |  | Valine | Valine interconversion/degradation | 2.6.1.42 |
|  |  | Glutamic acid | Valine biosynthesis | 2.6.1.42 |
| Leucine | | Pyruvic acid | Leucine biosynthesis | 2.6.1.42 |
|  |  | Leucine | Leucine  interconversion/degradation | 2.6.1.42 |
|  |  | Glutamic acid | Leucine biosynthesis | 2.6.1.42 |
| Alanine | | Pyruvic acid | Alanine biosynthesis | 2.6.1.2 |
|  |  | Alanine | Alanine  interconversion/degradation | 2.6.1.2 |
|  |  | Glutamic acid | Alanine biosynthesis | 2.6.1.2 |
|  |  | Valine | Alanine biosynthesis | 2.6.1.66 |
| Citric acid | | Pyruvic acid | Citric acid biosynthesis | 1.2.4.1  2.3.3.8 |
|  |  | Citric acid | Citric acid  interconversion/degradation | 4.2.1.3 |
|  |  | Malic acid | Citric acid biosynthesis | 1.2.4.1  2.3.3.8 |
| Aspartic acid | | Aspartic acid | Aspartic acid  interconversion/degradation | 6.3.5.4  2.7.2.4  4.2.3.1 |
|  |  | Malic acid | Aspartic acid biosynthesis | 1.1.1.37  2.6.1.1 |
|  |  | Glutamic acid | Aspartic acid biosynthesis | 2.6.1.1 |
|  |  | Glutamine | Asparagine biosynthesis | 6.3.5.4 |
| Asparagine | | Aspartic acid | Asparagine biosynthesis | 6.3.5.4 |
|  |  | Asparagine | Asparagine  interconversion/degradation | 3.5.1.1 |
|  |  | Glutamine | Asparagine biosynthesis | 6.3.5.4 |
| Threonine | | Aspartic acid | Threonine biosynthesis | 4.2.3.1 |
|  |  | Threonine | Threonine  interconversion/degradation | 4.3.1.19 |
| Succinic acid | | 2-Oxoglutaric acid | Succinic acid biosynthesis | 6.2.1.5 |
|  |  | Succinic acid | Succinic acid  interconversion/degradation | 1.3.5.1 |
|  |  | Putrescine | Succinic acid biosynthesis | 1.4.3.10  1.2.1.19 |
| Fumaric acid | | Succinic acid | Fumaric acid biosynthesis | 1.3.5.1 |
|  |  | Fumaric acid | Fumaric acid  interconversion/degradation | 4.2.1.2 |
| Malic acid | | Fumaric acid | Malic acid biosynthesis | 4.2.1.2 |
|  |  | Malic acid | Malic acid  interconversion/degradation | 1.1.1.37 |
|  |  | Glutamic acid | Aspartic acid biosynthesis | 2.6.1.1 |
| Proline | | Proline | Proline  interconversion/degradation | 1.5.99.8 |
|  |  | Glutamic acid | Proline biosynthesis | 2.7.2.11  1.2.1.41  1.5.1.2 |

| **Jacobian Entry**  **df[..]/d[..]** | | **Associated Pathway** | **Representative**  **Enzyme Reaction**  **(EC Number)** |
| --- | --- | --- | --- |
| **Function of**  **(df[..])** | **Variable**  **d[]** |  |  |
| Putrescine | Putrescine | Putrescine  interconversion/degradation | 2.5.1.16  1.4.3.10 |
|  | Spermidine | Spermidine  interconversion/degradation | 1.5.3.17 |
|  | Glutamic acid | Putrescine biosynthesis | 4.1.1.19  3.5.3.11 |
| Spermidine | Putrescine | Spermidine biosynthesis | 2.5.1.16 |
|  | Spermidine | Spermidine  interconversion/degradation | 1.5.3.17  2.5.1.22 |
| 2-Oxoglutaric acid | Pyruvic acid | Valine/Leucine biosynthesis | 2.6.1.42 |
|  | Glutamic acid | Glutamic acid  interconversion/degradation | 2.6.1.2  2.6.1.1  2.6.1.4  2.6.1.79 |
|  | Citric acid | 2-Oxoglutaric acid biosynthesis | 4.2.1.3  1.1.1.41 |
|  | Malic acid | Aspartic acid biosynthesis | 2.6.1.1 |
|  | 2-Oxoglutaric acid | 2-Oxoglutaric acid interconversion/degradation | 1.4.1.13  1.4.1.2  6.2.1.5 |
| Glutamic acid | Serine | Tryptophan biosynthesis | 4.2.1.122  4.1.3.27 |
|  | Pyruvic acid | Valine/Leucine/Alanine biosynthesis | 2.6.1.42  2.6.1.2 |
|  | Aspartic acid | Asparagine biosynthesis | 6.3.5.4 |
|  | Malic acid | Aspartic acid biosynthesis | 2.6.1.1 |
|  | Glutamic acid | Glutamic acid  interconversion/degradation | 2.6.1.42  2.6.1.2  6.3.5.4  2.6.1.1  2.6.1.4  2.6.1.79  1.3.1.78  2.7.2.11  4.1.1.19  3.5.3.11 |
|  | Glutamine | Tryptophan/Asparagine  biosynthesis | 6.3.5.4  4.1.3.27 |
|  | 2-Oxoglutaric acid | Glutamate biosynthesis | 1.4.1.13  1.4.1.2 |
| Glutamine | Serine | Tryptophan biosynthesis | 4.1.3.27 |
|  | Aspartic acid | Asparagine biosynthesis | 6.3.5.4 |
|  | Glutamic acid | Glutamine biosynthesis | 6.3.1.2 |
|  | Glutamine | Glutamine  interconversion/degradation | 4.1.3.27  6.3.5.4  3.5.1.2 |
