# Supplementary material for: Eco-Metabolomics and Metabolic Modeling: Making the Leap From Model Systems in the Lab to Native Populations in the Field
Source: Front Plant Sci. 2018 Nov 6;9:1556. doi: 10.3389/fpls.2018.01556 (PMC6232504; doi:10.3389/fpls.2018.01556)
Supplement: PRESENTATION S2 — Metabolite – GPS – correlation network. [file Presentation_2.PPTX]

## Slide 1
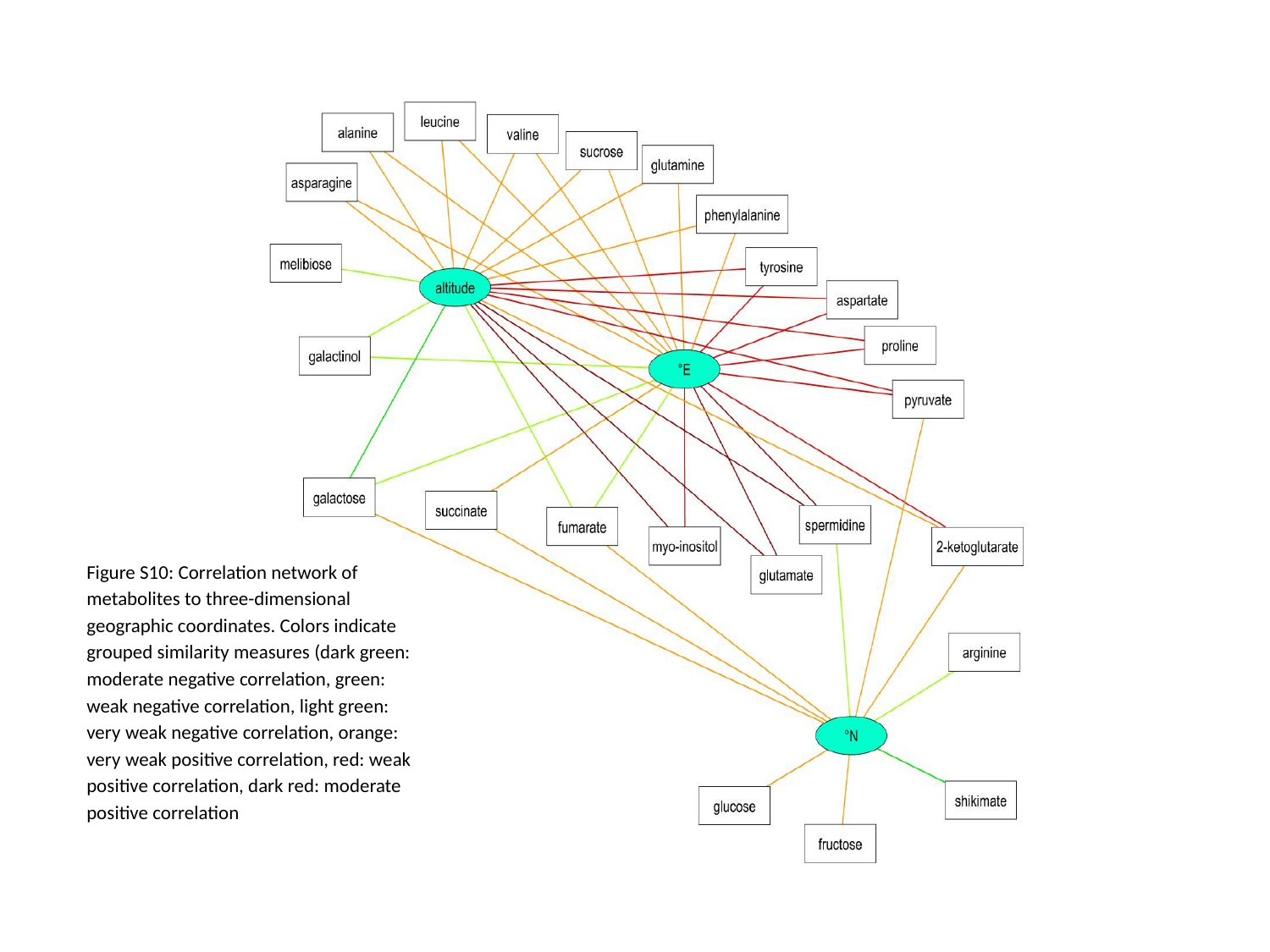

Figure S10: Correlation network of metabolites to three-dimensional geographic coordinates. Colors indicate grouped similarity measures (dark green: moderate negative correlation, green: weak negative correlation, light green: very weak negative correlation, orange: very weak positive correlation, red: weak positive correlation, dark red: moderate positive correlation
